# Supplementary material for: Structural basis for human chondroitin sulfate chain polymerization
Source: Nat Commun. 2025 Nov 26;16:11663. doi: 10.1038/s41467-025-66787-5 (PMC12748766; doi:10.1038/s41467-025-66787-5)

Supplementary information for

Structural basis for human chondroitin sulfate chain polymerization

Poushalee Dutta[1], Rosa Lorizolla Cordeiro[1], Mélanie Friedel-Arboleas[1], Marie Bourgeais[1], Sylvain D. Vallet[1], Margot Weber[1], Margaux Molinas[1], Huazhang Shu[2], Magnus N. N. Grønset[2], Rebecca L. Miller[2], Elisabetta Boeri Erba[1], Rebekka Wild[1,#]

[1]Institut de Biologie Structurale, UMR 5075, University Grenoble Alpes, CNRS, CEA, 38000 Grenoble, France.

[2]Copenhagen Center for Glycocalyx Research, Department of Cellular and Molecular Medicine, Faculty of Health Sciences, University of Copenhagen; Blegdamsvej 3, DK-2200 Copenhagen N, Denmark.

[#]Correspondence: rebekka.wild@ibs.fr

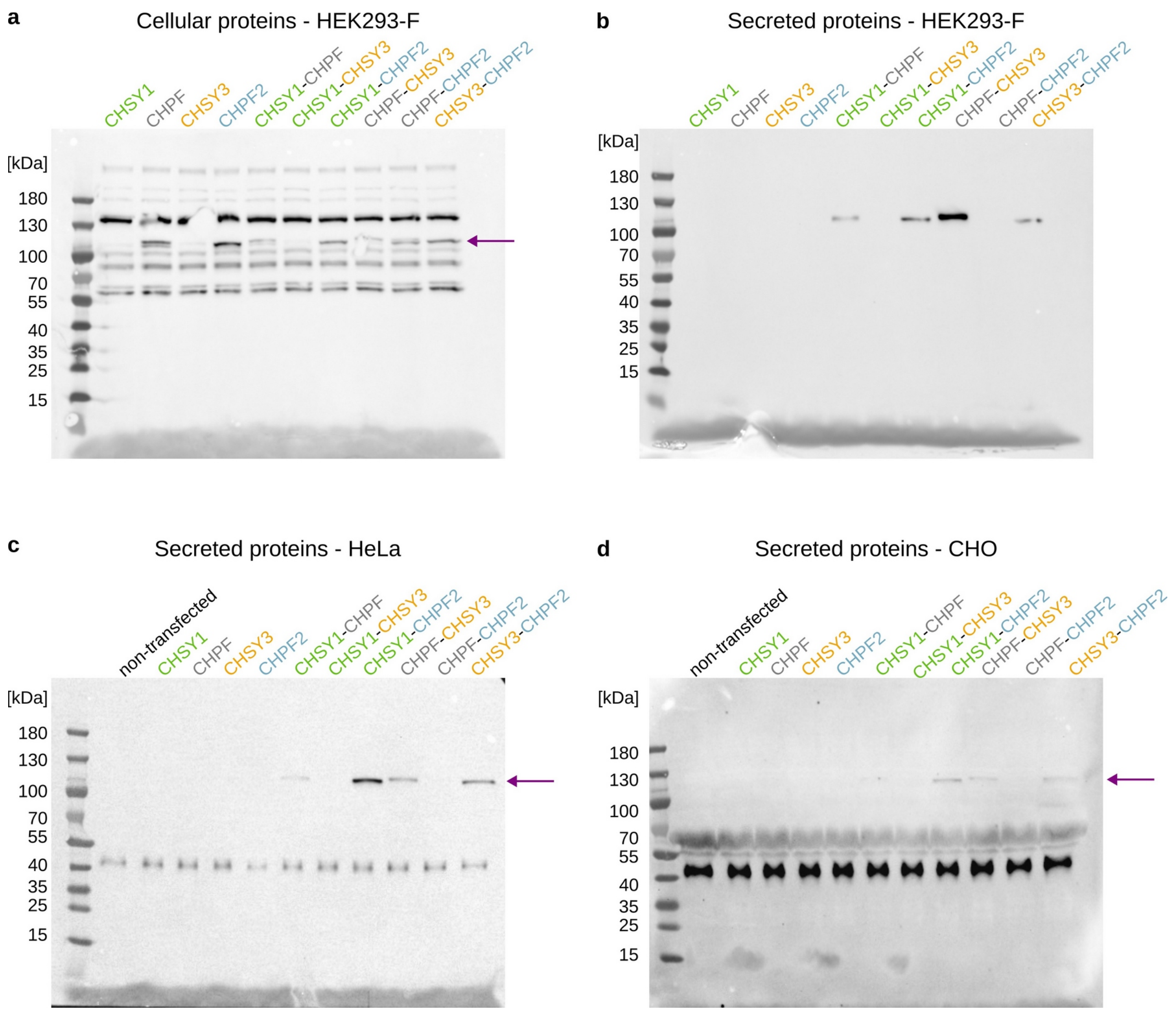

Supplementary Figure 1: Western blot analysis of transient expression of CS polymerase enzymes in different cell types.

(a) Analysis of cellular protein content following transfection of CS synthase-encoding constructs containing an N-terminal secretion signal for targeting proteins to the cell medium, using human embryonic kidney Freestyle 293-F (HEK293-F) cells. His-tagged proteins were detected using an anti-His HRP-conjugated antibody and a chemiluminescent signal recorded for 0.3s. **(b)** Detection of His-tagged, secreted proteins in the cell medium upon transfection of HEK293-F cells using a 2s recording time. **(c)** Analysis of proteins secreted into the medium upon transfection of HeLa cells. The chemiluminescent signal was recorded for 59s. **(d)** Detection of proteins in the cell medium following transfection of Chinese hamster ovary (CHO) cells using a 120 s exposure time. Purple arrows indicate bands corresponding to the expected molecular weight of 103 kDa for secreted CHSY1 (aa68-aa802), 100 kDa for CHPF (aa81-aa775), 102 kDa for CHSY3 (aa157-aa882), and 101 kDa for CHPF2 (aa57-aa772). Uncropped blots from single experiments (n=1) are shown at the end of this supplementary information file.

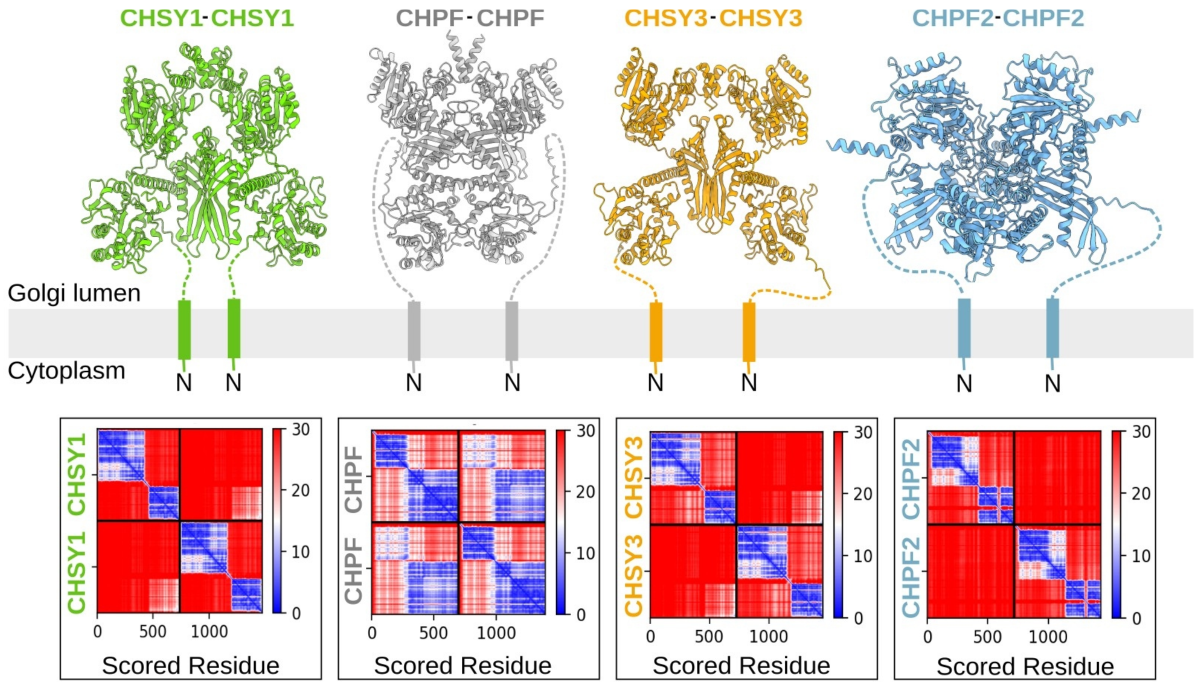

Supplementary Figure 2: CS synthase proteins do not seem to form homodimeric complexes. AlphaFold 2 predicted models for the four homodimeric CS polymerase complexes[1,2]. The models are shown in cartoon representation with CHSY1 in green, CHPF in grey, CHSY3 in orange, and CHPF2 in light blue. The N-terminal anchoring helices and flexible stem regions (dotted lines), which were omitted during model prediction, were drawn by hand. The corresponding predicted aligned error matrices are shown below.

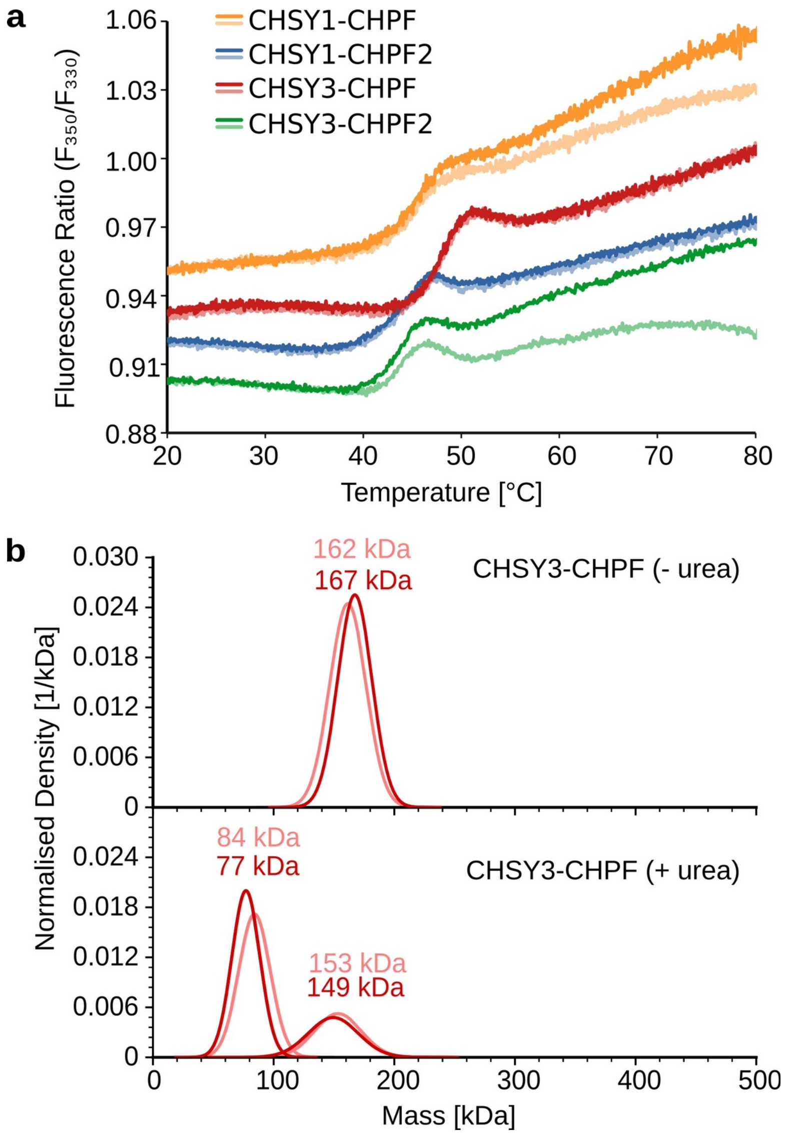

Supplementary Figure 3: Biophysical characterization of wild-type CS polymerase complexes.
(a) Fluorescence curves of nano-differential scanning fluorimetry (nanoDSF) measurements for wild-type CS polymerase complexes. **(b)** Mass photometry analysis of CHSY3-CHPF complex before and after 5.4 M urea treatment, with corresponding masses indicated alongside. The expected masses are 165 kDa for the CHSY3-CHPF complex, 86 kDa for CHSY3, and 79 kDa for CHPF. Additional experimentally derived values are summarized in Supplementary Table 3. Source data are provided as a Source Data file.

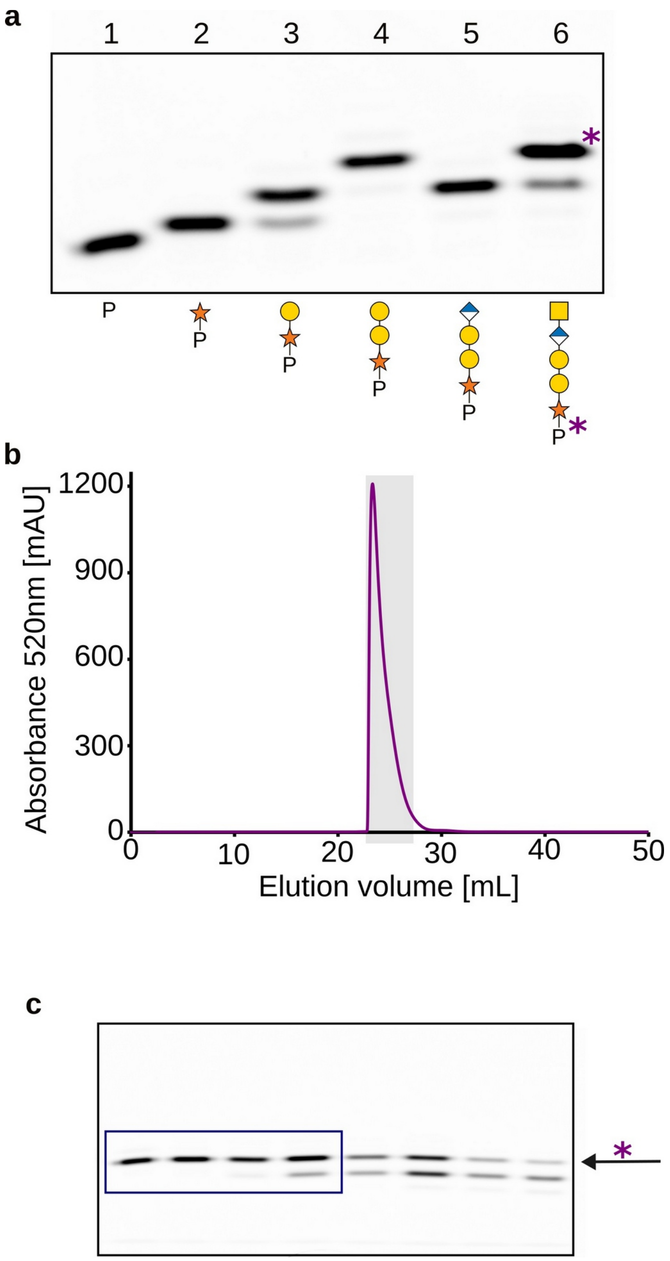

Supplementary Figure 4: Step-wise addition of pentsaccharide onto fluorescent CSF1 peptide.
(a) FACE analysis of reaction products from chemo-enzymatic synthesis of pentasaccharide onto fluorescent CSF1 peptide. Lane 1 contains the fluorescent CSF1 peptide before glycan addition, and lanes 2-6 show the peptide after mono-, di-, tri-, tetra-, and pentasaccharide addition, respectively. Each glycan addition is catalyzed by a distinct enzyme. Reaction products were visualized using a fluorescence imager, and glycan addition can be followed by shifts in migration speed. Monosaccharide symbols follow the symbol nomenclature for glycans (SNFG) system[3]. **(b)** The pentasaccharide peptide generated in (a) was purified by size exclusion chromatography (SEC). Absorbance is indicated in milli-absorbance units (mAU). **(c)** Peak fractions from SEC (highlighted in grey) were further analyzed by FACE. A purple asterisk indicates the pentasaccharide peptide product, and fractions corresponding to lanes marked by a blue box were pooled. Source data are provided as a Source Data file.

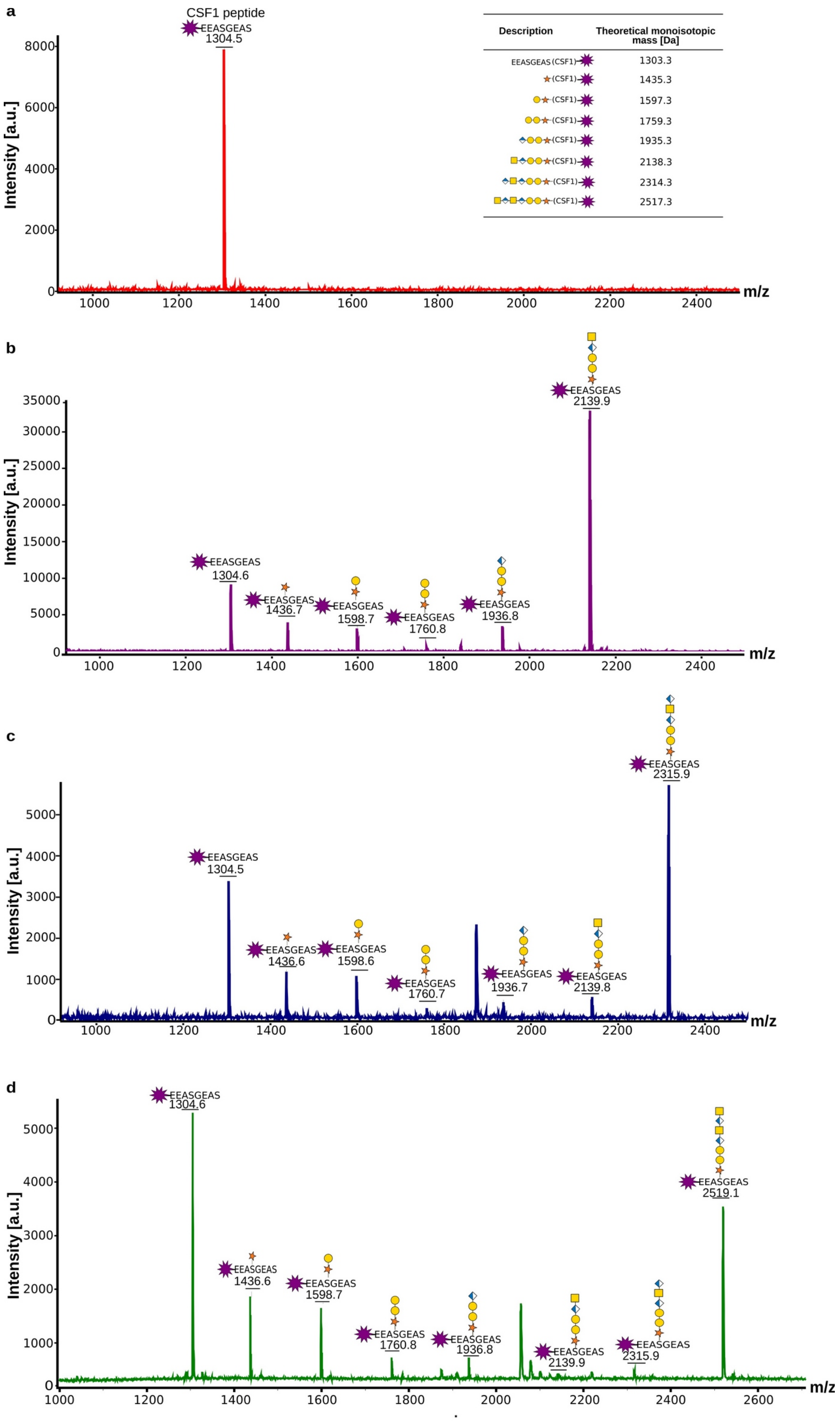

Supplementary Figure 5: Mass spectrometry analysis of Penta-CSF1 and Hexa-CSF1 peptides.
(a) A synthetic fluorescent peptide (TAMRA-EEASGEAS) was derived from the chondroitin sulfate proteoglycan colony stimulating factor 1 (CSF1). This peptide was used as a control for MALDI-TOF analyses. Data are shown as mass-to-charge ratio (m/z). Since all ions are singly protonated, the mass of each peptide can be obtained from the m/z value by subtracting the mass of one proton (1.0 Da). The theoretical monoisotopic masses of the peptides are summarized in a table. **(b)** MS spectrum of purified Penta-CSF1 peptide carrying a pentasaccharide linker (GalNAc-GlcA-Gal-Gal-Xyl). Traces of reaction byproducts are present as well. **(c)** MS spectrum of purified Hexa-CSF1 peptide with a hexasaccharide linker (GlcA-GalNAc-GlcA-Gal-Gal-Xyl). **(d)** MS spectrum of purified Hepta-CSF1 peptide with a heptasaccharide linker (GalNAc-GlcA-GalNAc-GlcA-Gal-Gal-Xyl). Graphs show results from a single experiment (n=1). Source data are provided as a Source Data file.

a

b

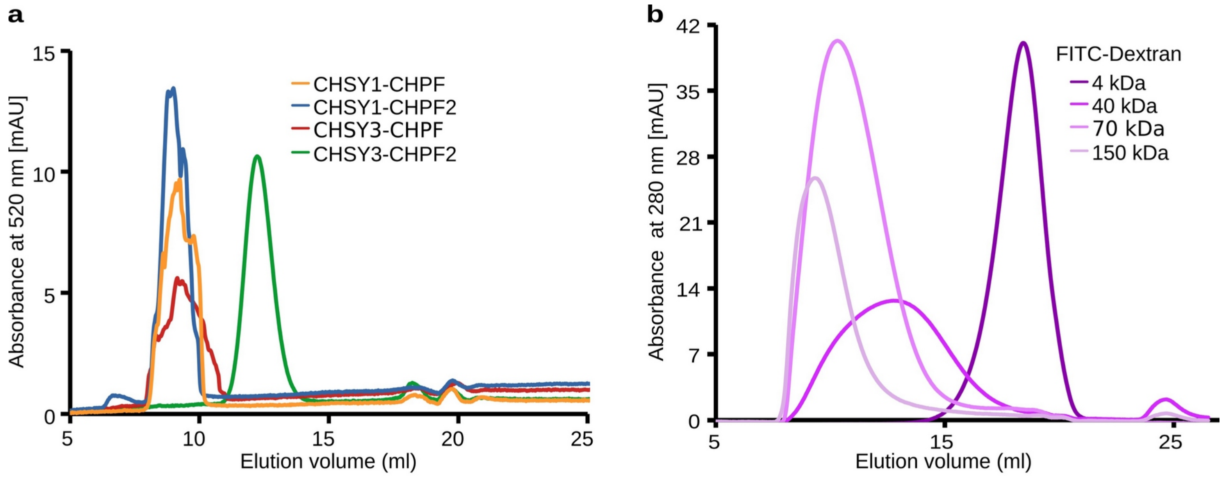

c

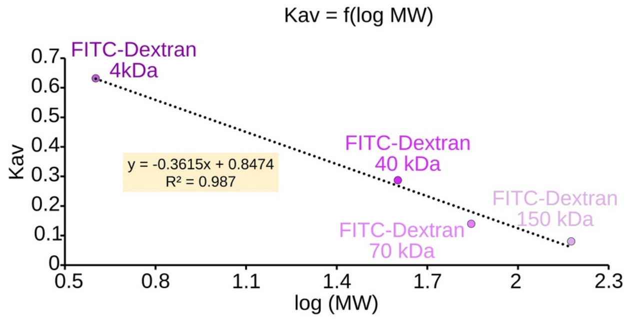

$Kav = (V_e - V_0) / (V_t - V_0)$
V_e = elution volume
V_0 = void volume (blue dextran)
V_t = total bed volume (imidazole)

d

Standards/Products	MW (kDa)	log MW	V_e (mL)	Kav
FITC-Dextran 4 kDa	4	0.60	18.42	0.63
FITC-Dextran 40 kDa	40	1.60	12.74	0.29
FITC-Dextran 70 kDa	70	1.84	10.31	0.14
FITC-Dextran 150 kDa	150	2.17	9.32	0.08
Blue dextran	2000	3.30	8	0
Imidazole	0.07	-1.17	24.48	1
CHSY1-CHPF	137	2.13	9.24	0.07
CHSY1-CHPF2	151	2.18	8.99	0.06
CHSY3-CHPF	142	2.15	9.14	0.07
CHSY3-CHPF2	43	1.63	12.23	0.26

Supplementary Figure 6: Size determination of chondroitin chains by size-exclusion chromatography
(a) Size-exclusion chromatography analysis of reaction products of different wild-type polymerase complexes using a Superdex 200 10/300 GL column, with a void volume of 8 mL. Absorbance of fluorescently labelled glycopeptide was detected at 520 nm. **(b)** Calibration of Superdex 200 10/300 GL column using commercial fluorescein isothiocyanate (FITC)-labelled dextran standards. **(c)** A calibration curve was calculated based on the peak maxima from chromatography analysis in (b). **(d)** Table summarizing molecular weights (MW), elution volumes (V_e), and the Kav constants for size-exclusion chromatography analysis of standards and CS polymerase reaction products. Source data are provided as a Source Data file.

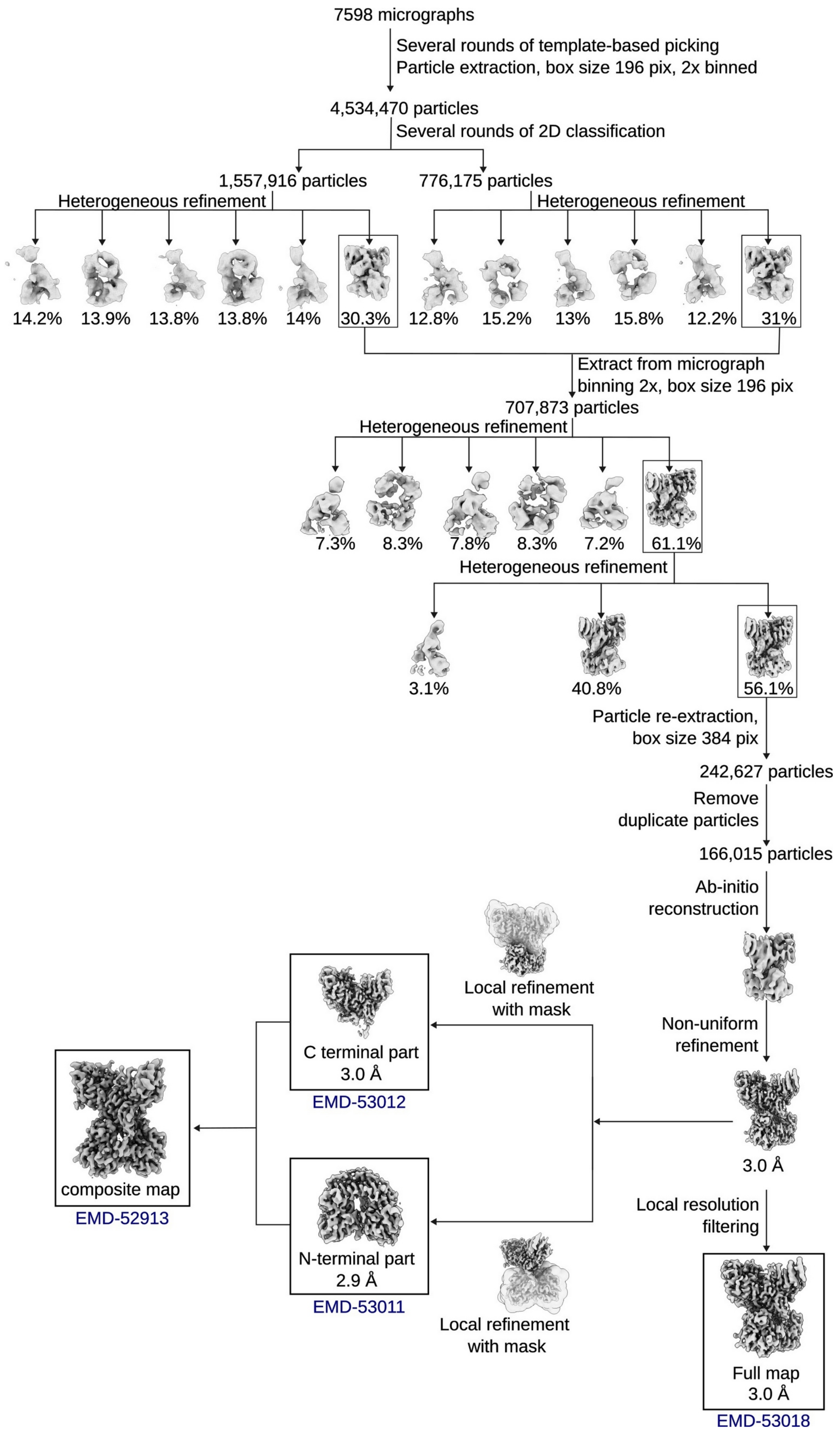

Supplementary Figure 7: Cryo-EM data processing flow chart.

EM data was processed using cryoSPRAC v3.3.1 software. The full consensus map, the N- and C-terminal maps obtained from focused refinements, and the generated composite map are accessible through the EMDB database. Corresponding accession codes are indicated.

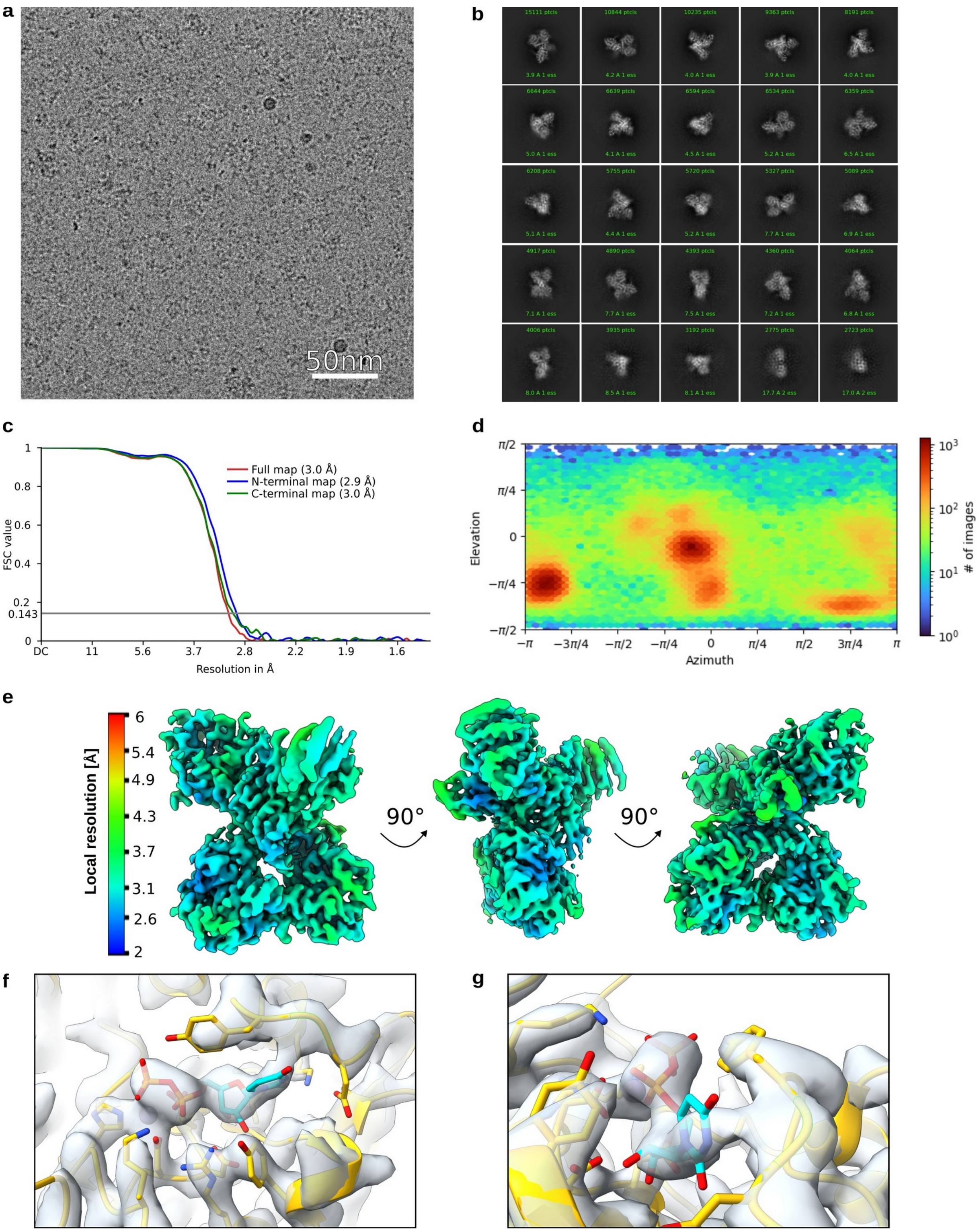

Supplementary Figure 8: EM data quality assessment.

(a) Exemplary motion-corrected and dose-weighted micrograph, from a total of 7598 micrographs. **(b)** 2D class averages of particles used for calculating the final map. **(c)** Fourier shell correlation (FSC) curve indicating estimated resolutions based on the FSC = 0.143 criterion as generated by cryoSPARC v3.3.1. Source data are provided as a Source Data file. **(d)** Angular distribution plot as generated by cryoSPARC v3.3.1. **(e)** Final local resolution-filtered EM map, colored by local resolution as estimated in phenix. The CS polymerase complex is shown from three orientations. **(f)** and **(g)** Map-to-model fit for UDP molecule in the GlcA-T domain of CHSY3 shown from two different orientations. The map contour level is 0.165.

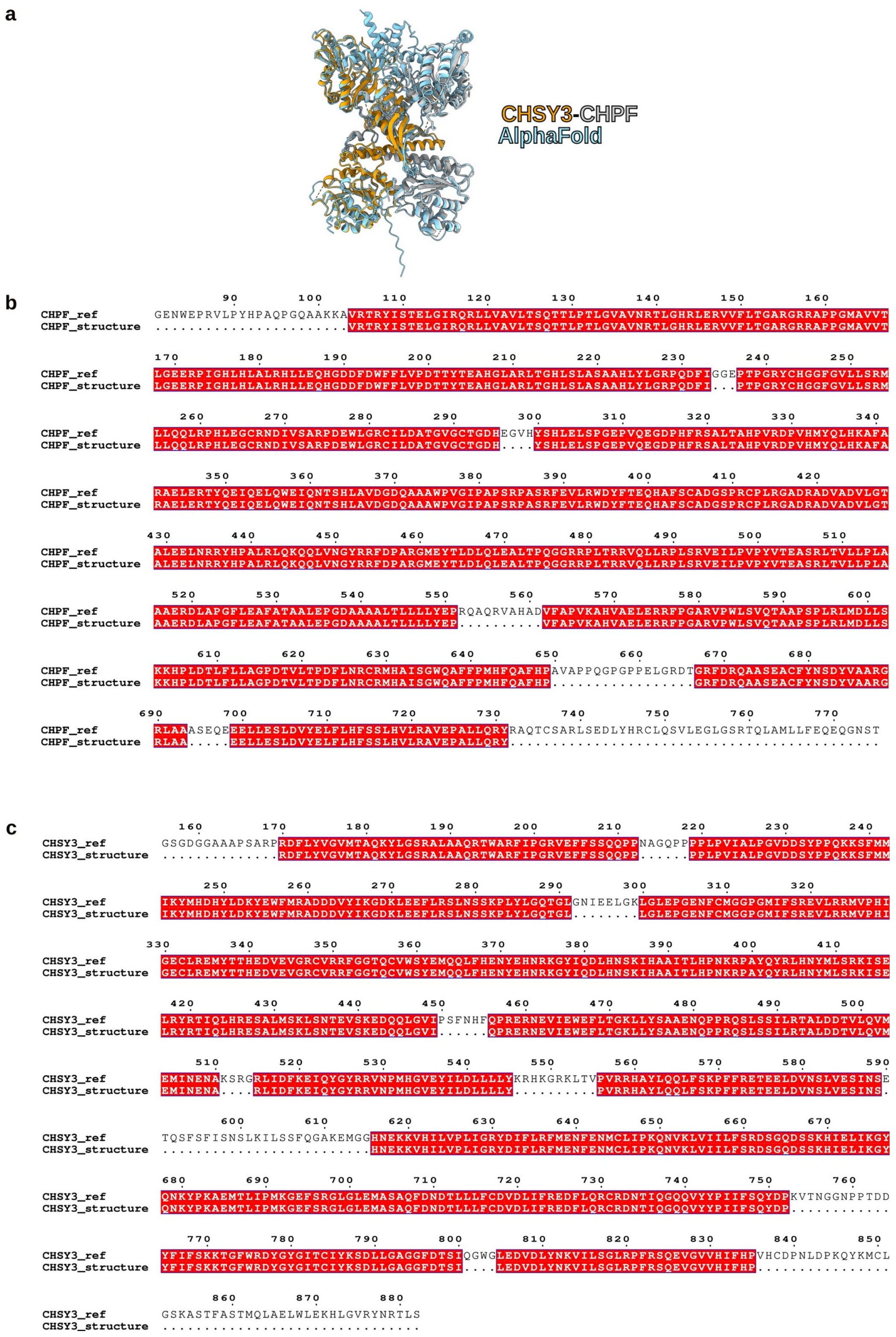

Supplementary Figure 9: Superposition of experimental and AlphaFold 2-predicted CHSY3-CHPF complex structure.

(a) Cryo-EM structure of the CHSY3-CHPF complex, colored in orange and grey, and the model predicted using AlphaFold 2, colored in blue, were superimposed using the matchmaker command in ChimeraX[4]. The root mean square deviation (RMSD) between 522 pruned atom pairs (Cα) is 1.080 Å. **(b)** and **(c)** Alignment of the native protein sequences (ref) of CHPF and CHSY3 with the residues observed in the cryo-EM structure. The gaps reflect flexible loops and the N- and C-termini that were not visible.

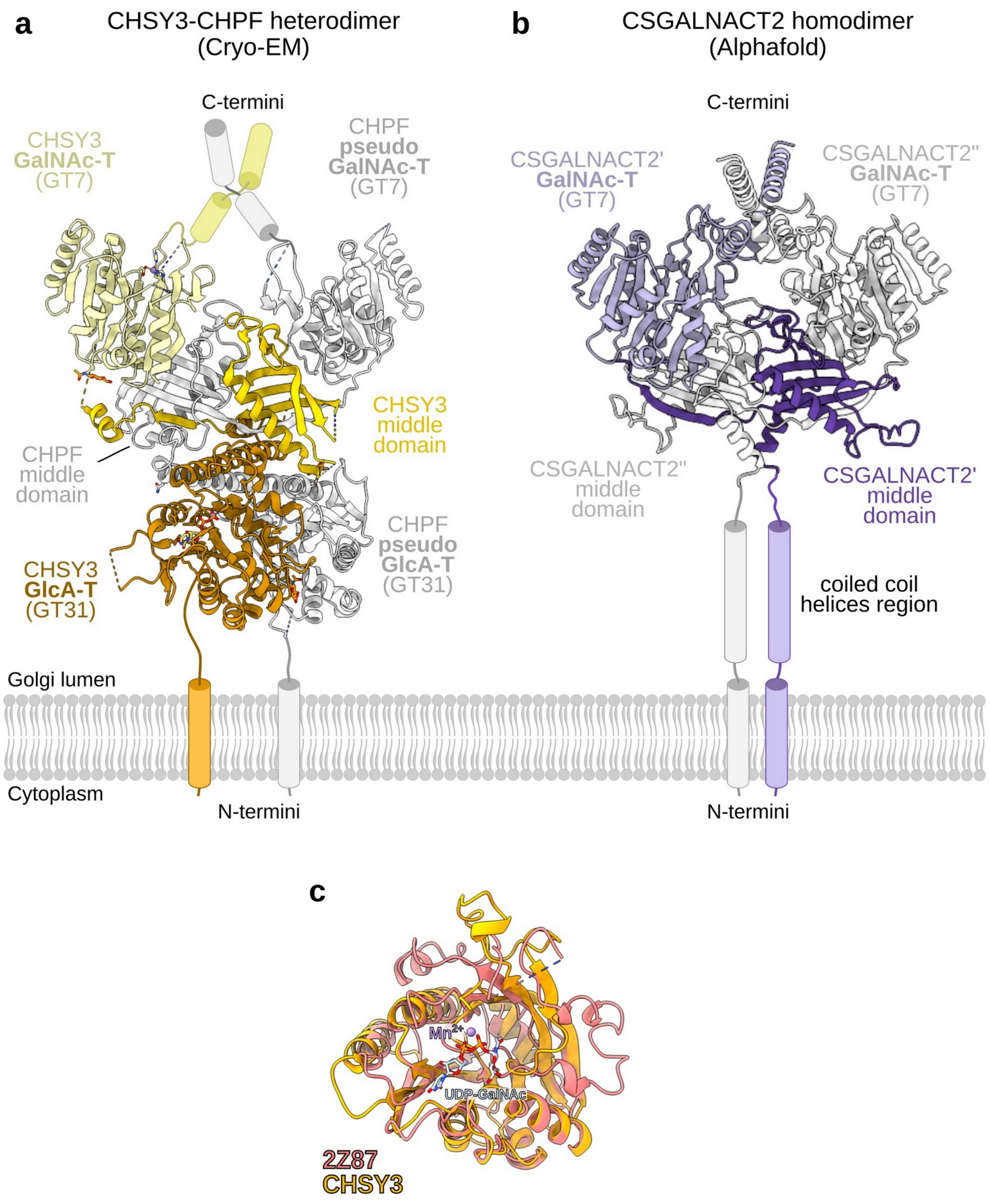

Supplementary Figure 10: Comparison of the GalNAc-T domain of CHSY3 with close structural homologs.

Side-by-side comparison of the experimental CHSY3–CHPF complex structure **(a)** and the AlphaFold-predicted homodimeric CSGALNACT2 complex **(b)**. The GalNAc-T domain and the middle domain of CHSY3 exhibit the same overall topology as those of CSGALNACT2. The two structures superimpose well with a root mean square deviation (RMSD) of 1.008 Å for 208 out of 389 residues (Cα). **(c)** Superposition of the C-terminal GalNAc-T domain of CHSY3 with the crystal structure of the close structural homolog Escherichia coli strain K4 chondroitin polymerase in complex with UDP-GalNAc (PDB ID: 2Z87). The two structures superimpose well with a root mean square deviation (RMSD) of 1.145 Å for 76 out of 286 residues (Cα).

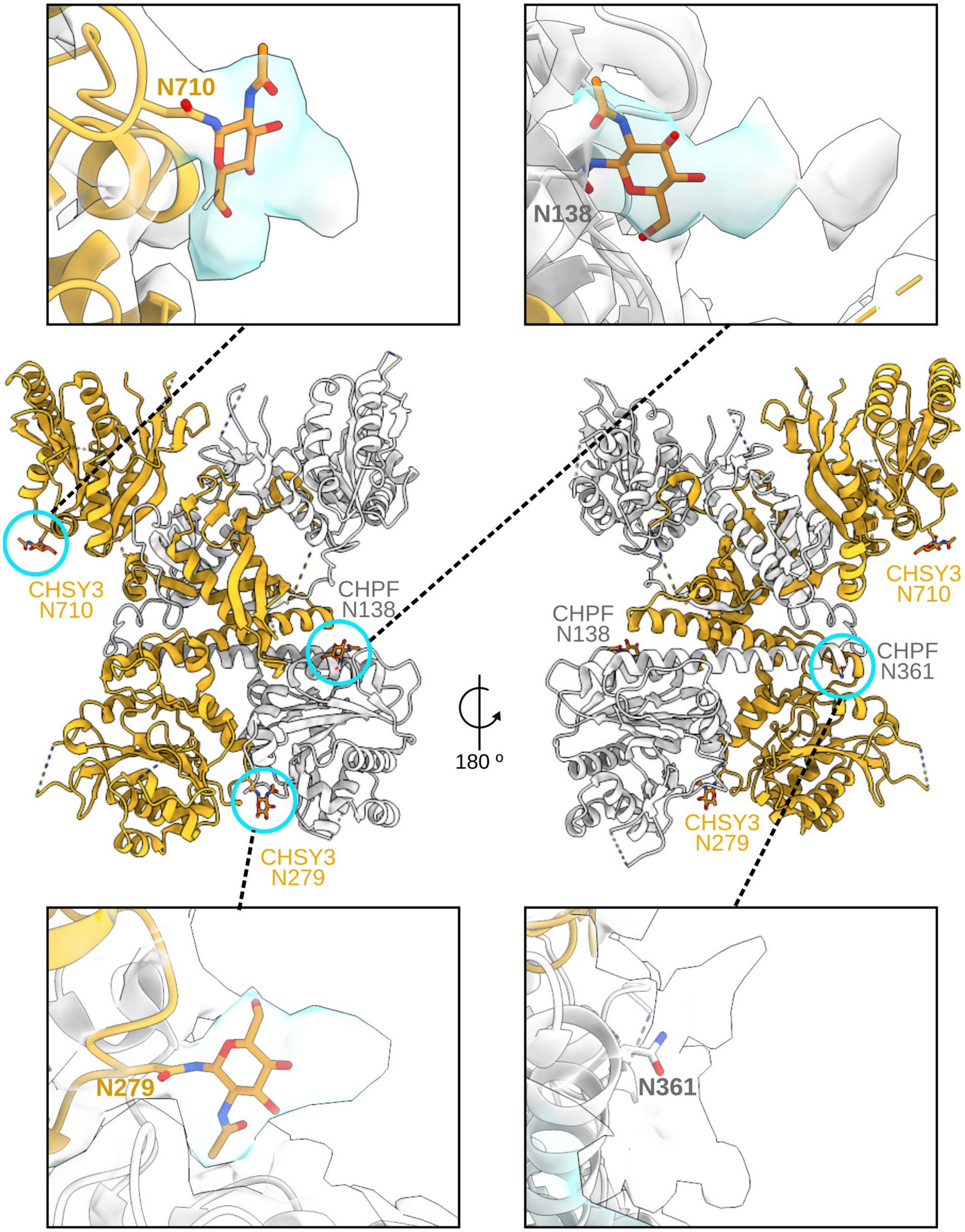

Supplementary Figure 11: EM map reveals *N*-glycosylation sites.

The EM map shows well-defined density for three N-linked glycans located at asparagine residues N279 and N710 of CHSY3, and N138 of CHPF. Additionally, weaker density near residue N361 of CHPF suggests the presence of a fourth N-glycosylation site. GlcNAc and asparagine residues corresponding to the glycosylation sites are represented in sticks. EM density in the close-up views is shown at a contour level of 0.0735.

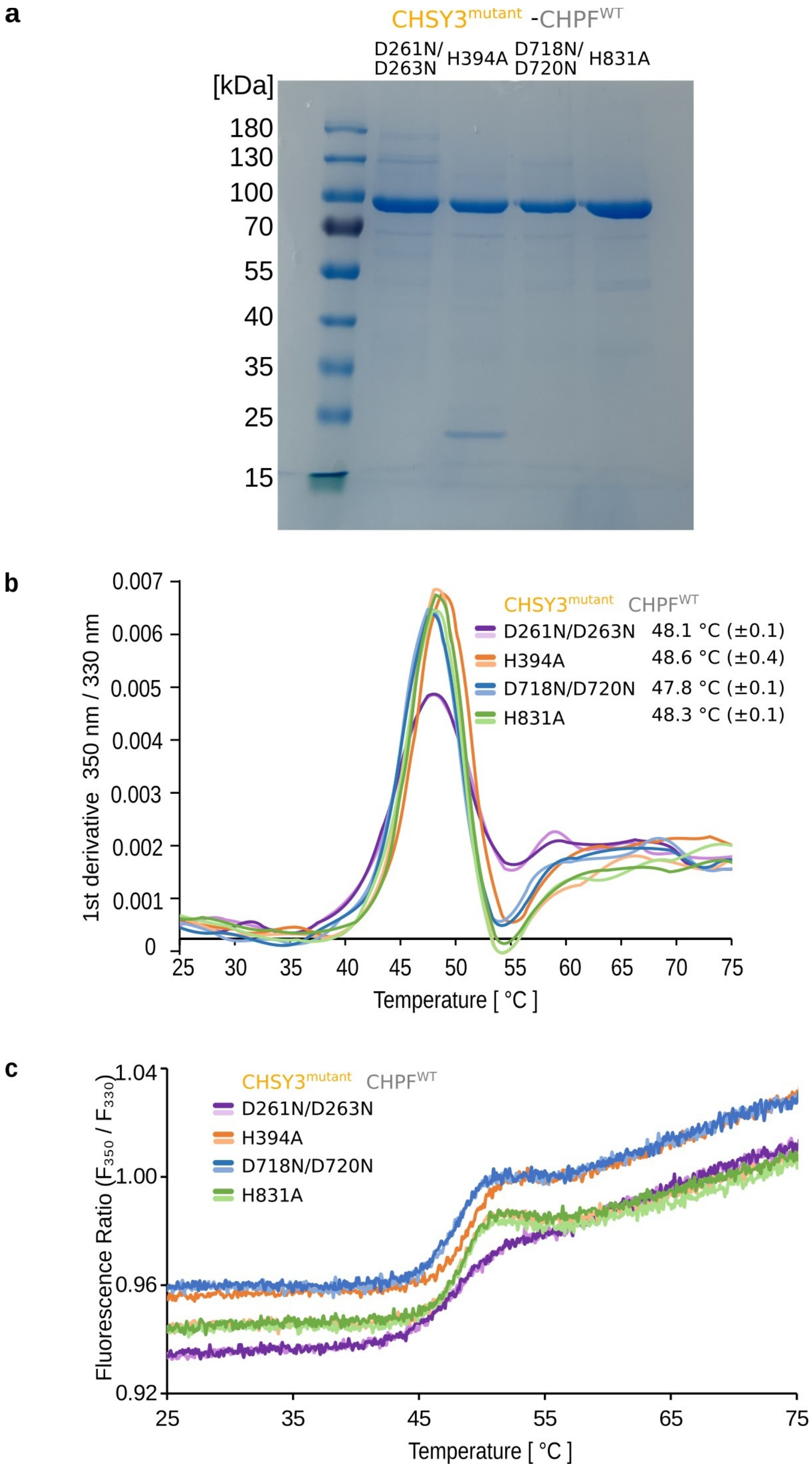

Supplementary Figure 12: Characterization of CHSY3-CHPF mutant complexes.
(a) Coomassie-stained SDS-PAGE analysis of the four CHSY3 mutant-containing CHSY3-CHPF complexes. **(b)** Thermal stability of mutant CHSY3-CHPF complexes was determined by nano-differential scanning fluorimetry (nanoDSF). The graph shows the first derivative plot of the fluorescence 350 nm/330 nm ratio. Average melting temperatures from duplicate measurements are indicated, with corresponding standard deviation in brackets. **(c)** Fluorescence curves of nanoDSF measurements for mutant CS polymerase complexes. Source data are provided as a Source Data file.

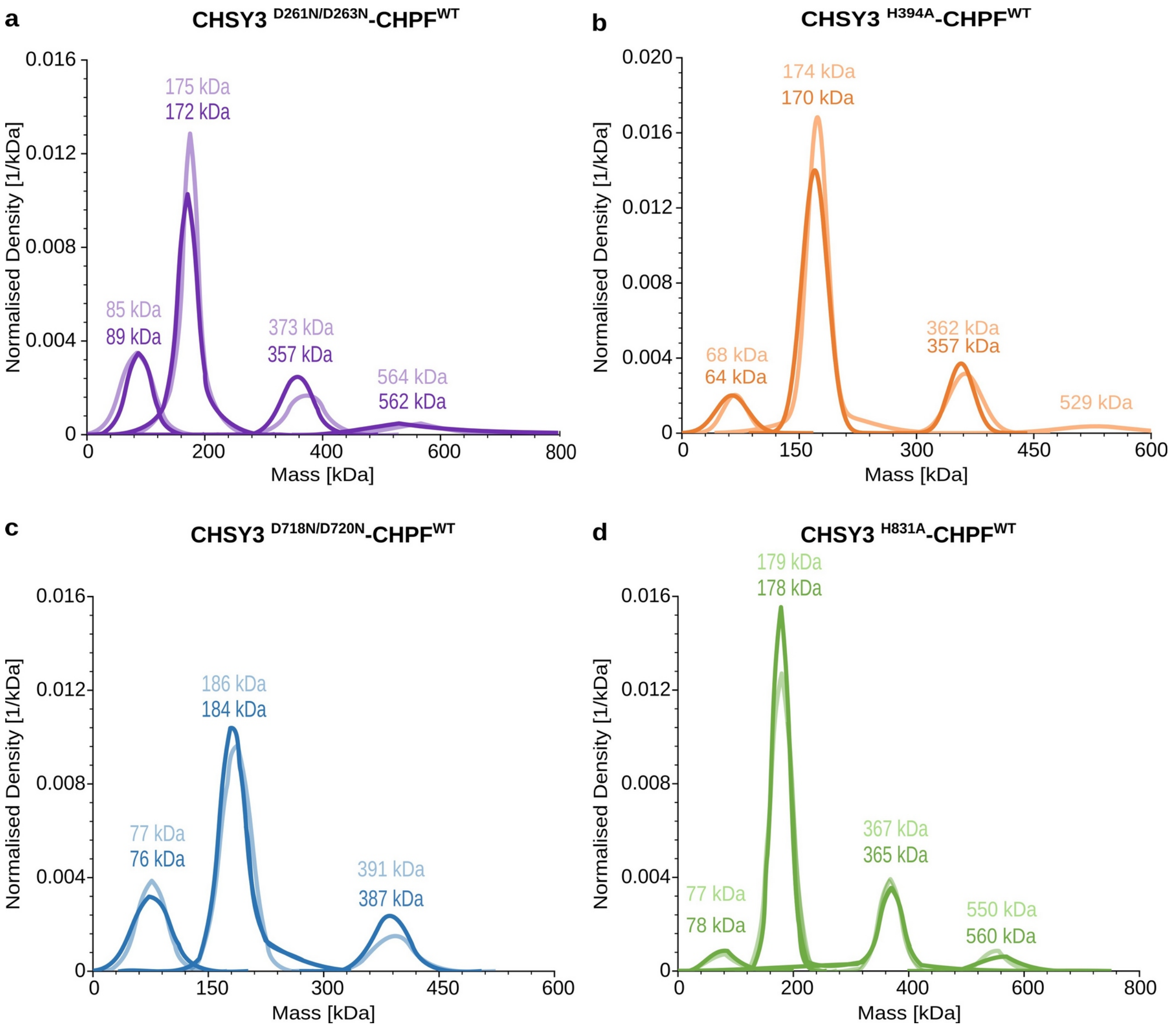

Supplementary Figure 13: Mass photometry analysis of mtant CHSY3–CHPF complexes.
(a-d) Mass photometry analysis of CHSY3 mutant-containing CHSY3–CHPF complexes. Measurements were performed in technical duplicates. The calculated molecular weights of the complexes are indicated, with an expected molecular weight of around 165 kDa for all CHSY3-CHPF complex variants. Source data are provided as a Source Data file.

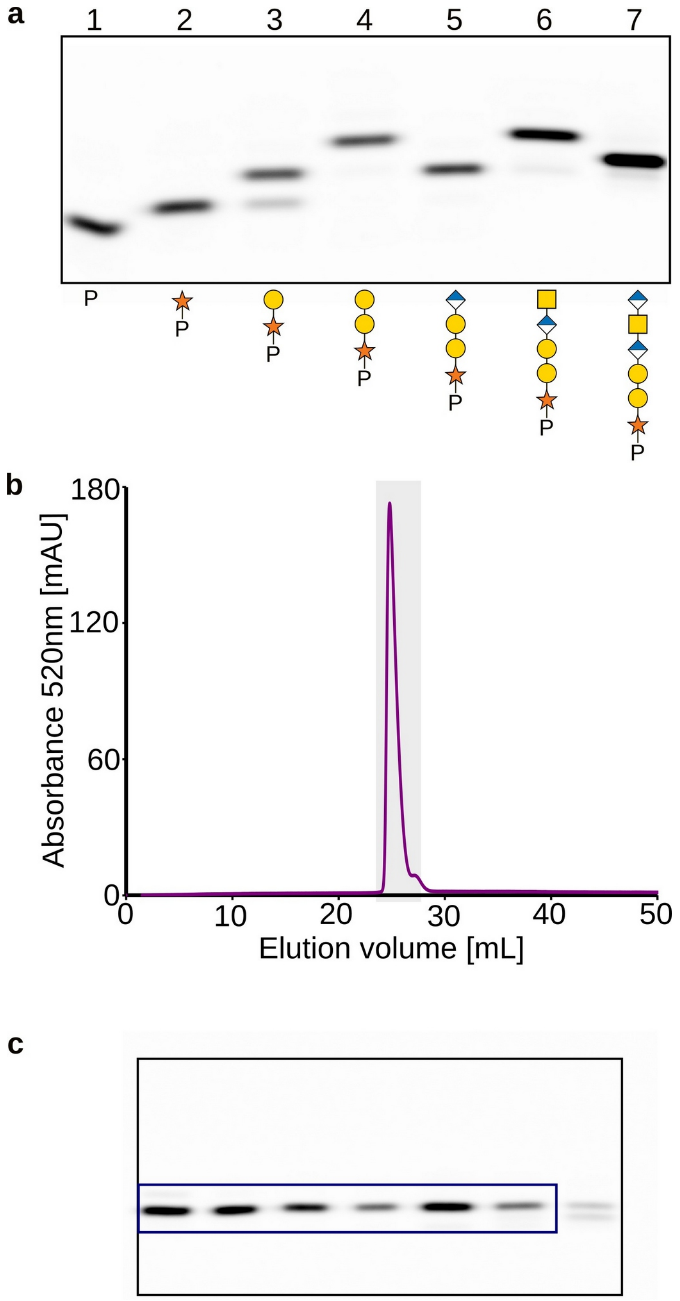

Supplementary Figure 14: Generation and purification of Hexa-CSF1 peptide.
(a) Chemo-enzymatic synthesis of hexasaccharide onto CSF1 peptide was analyzed by FACE. Lane 1 contains the fluorescent CSF1 peptide before glycan addition, and lanes 2-7 show the peptide after mono-, di-, tri-, tetra, penta-, and hexasaccharide addition, respectively. Reaction products were visualized using a fluorescence imager, and glycan addition can be followed by shifts in migration speed. Monosaccharide symbols follow the symbol nomenclature for glycans (SNFG) system[3]. **(b)** Hexa-CSF1 peptide was purified using SEC. (**c**) Peak fractions from SEC (highlighted in grey) were further analyzed by FACE. Hexasaccharide peptide-containing fractions, marked by a blue box, were pooled. Source data are provided as a Source Data file.

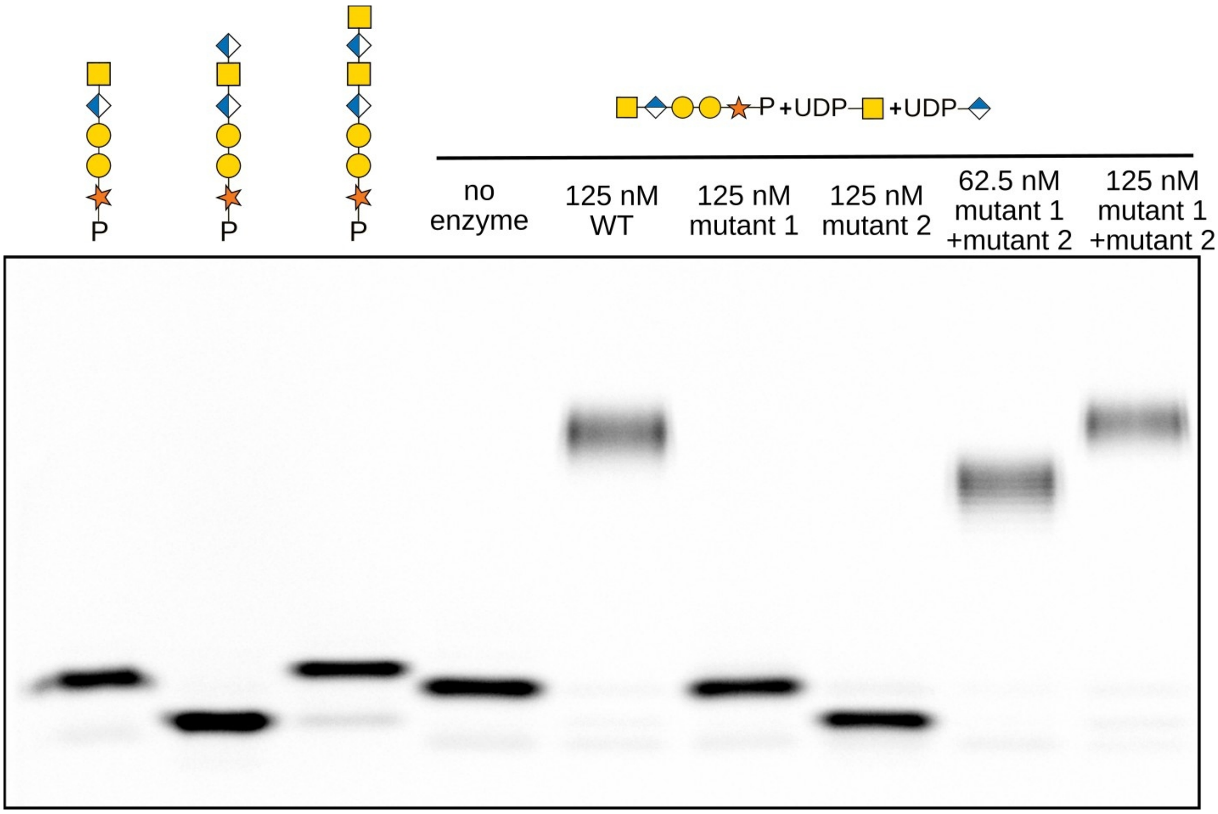

Supplementary Figure 15: Rescue experiment of mutant CHSY3-containing CS polymerase complexes. Reactions containing wild-type (WT) or CHSY3 mutant-containing CHSY3/CHPF complexes, the Penta-CSF1 acceptor substrate, and both UDP-GalNAc and UDP-GlcA donor substrates were prepared. The ability of GlcA-T and GalNAc-T mutants to complement each other and thus restore chain elongation was tested. Reaction products were analyzed by FACE. Penta-, hexa-, and heptasaccharide peptides were used as molecular weight markers. Source data are provided as a Source Data file.

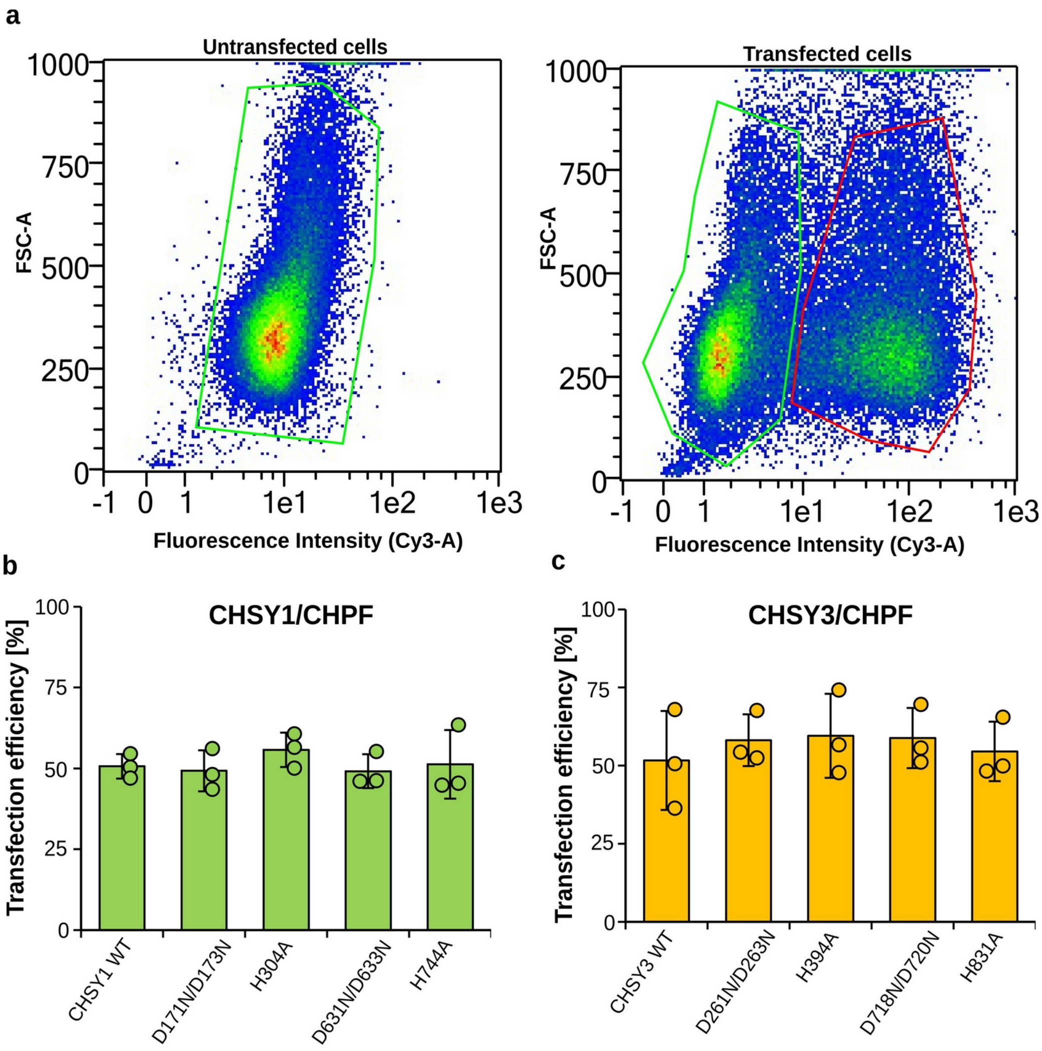

Supplementary Figure 16: Measurement of transfection efficiency by flow cytometry.
(a) Exemplary scatter dot plots of flow cytometry experiments using an anti-FLAG primary antibody and a Cy3 secondary antibody. The left panel shows the pattern of a cell population that was not transfected, and the right panel shows the pattern upon transfection with FLAG-CHSY3 and FLAG-CHPF encoding plasmids. Areas in the dot plot corresponding to untransfected and transfected cells are highlighted in green and red, respectively. **(b)** and **(c)** Bar plots showing the average transfection efficiency, calculated based on the number of successfully transfected cells (red box in a) in relation to the total number of analyzed cells. Error bars show standard deviation for mean values from three independent experiments (n=3). Source data are provided as a Source Data file.

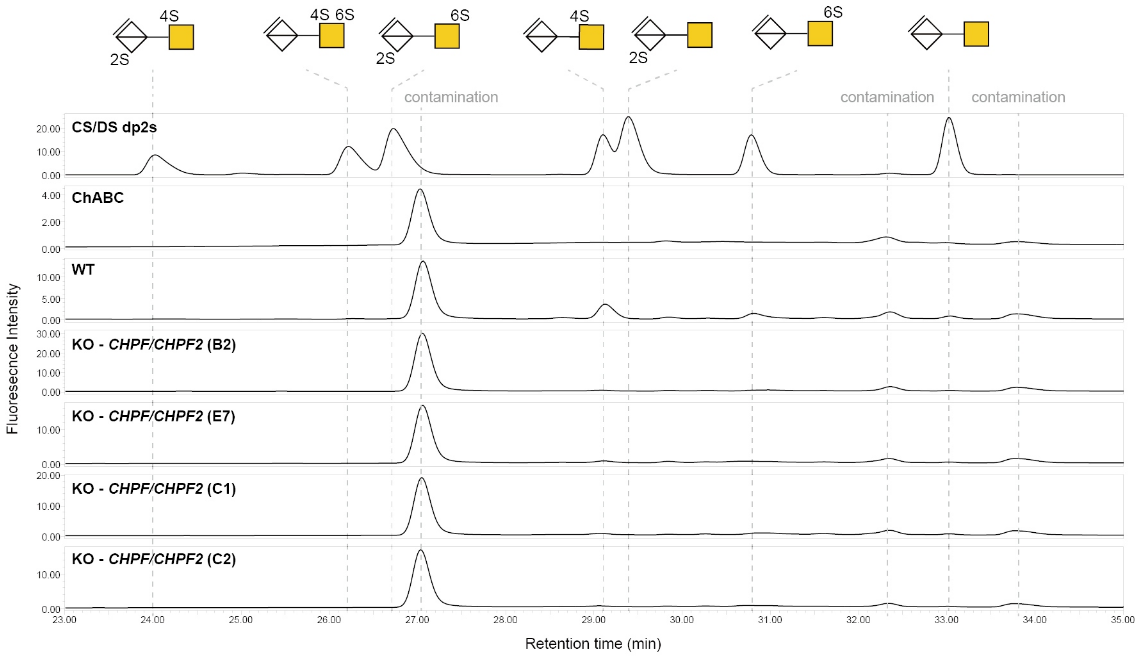

Supplementary Figure 17: HEK293 cells with *CHFP/CHPF2* double gene knock-out demonstrate no detectable chondroitin sulfate. RP-UPLC disaccharide analysis of purified GAGs from HEK293 WT and HEK293 *CHPF/CHPF2* KO total cell lysates. The top row shows peaks for labeled disaccharide standards (CS/DS dp2s, 20 pmol injection for each). The second row shows peaks that appear under chondroitinase ABC (ChABC) treatment without GAG samples, which were labelled as contamination. The CS/DS content of HEK293 WT cells was estimated to be ~62.6% 4-O-sulfated disaccharide (D0a4), ~28.0% 6-O-sulfated disaccharides (D0a6), ~6.8% non-sulfated disaccharide (D0a0), ~0.9% 2,4-di-sulfated disaccharide (D2a4), and ~1.6% 4,6-di-sulfated disaccharides (D0a10). 2-O-sulfated disaccharide (D2a0) and 2,6-di-sulfated disaccharides (D2a6) were not detectable. Four different *CHPF/CHPF2* (B2/E7/C1/C2) double gene KO clones were isolated, and chondroitin sulfate disaccharide analysis was performed. In the HEK293 CHPF/CHPF2 KO clones (B2/E7/C1/C2), chondroitin sulfate was not detectable. The analysis was repeated in technical duplicates. Source data are provided as a Source Data file.

a

Identity matrix [%]

	CHSY1	CHSY3	CHPF	CHPF2
CHSY1	100			
CHSY3	68.31	100		
CHPF	23.73	24.59	100	
CHPF2	24.61	24.2	59.15	100

b

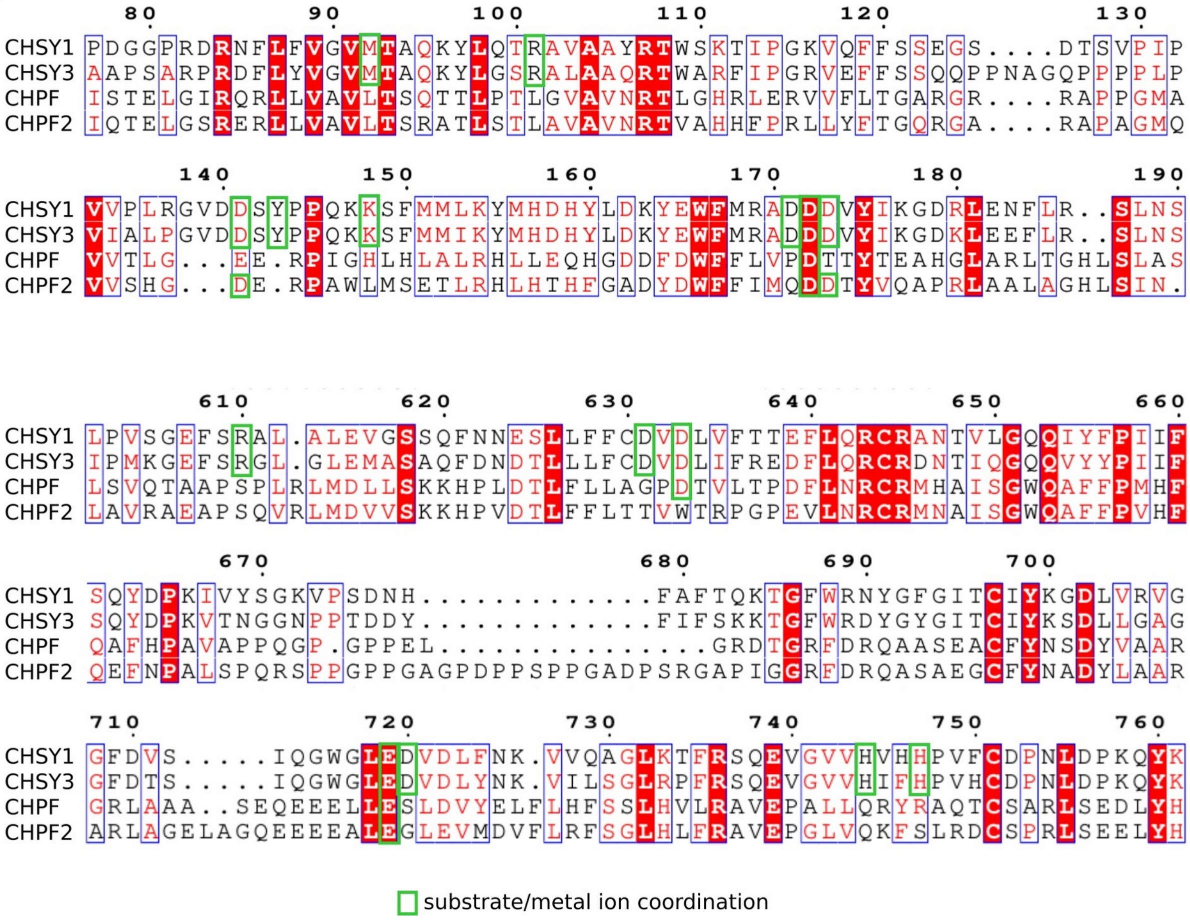

Supplementary Figure 18: Sequence analysis of human CS synthase proteins.
(a) Sequence identity between full-length human CS synthase proteins CHSY1, CHSY3, CHPF, and CHPF2 was calculated using Clustal Omega[5] and is displayed as an identity matrix. **(b)** Sequence alignment of catalytic regions of CHSY1, CHSY3, CHPF, and CHPF2 was performed in ESPript 3.0[6]. Residues involved in UDP and Mn^{2+} coordination are highlighted in green.

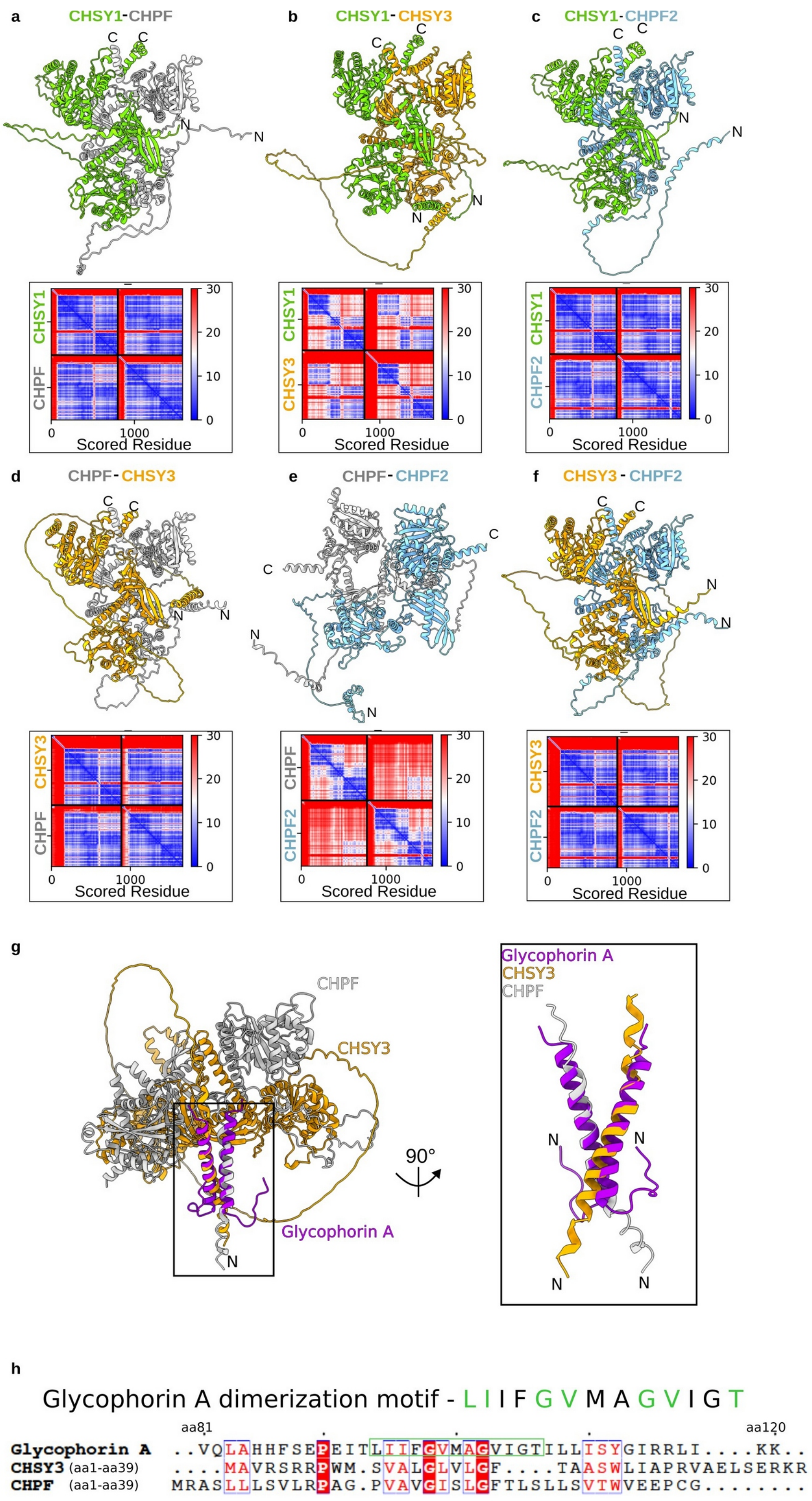

Supplementary Figure 19: Search for interactions in the N-terminal region of CS polymerase complexes. **(a–f)** AlphaFold2-predicted models of the full-length heterodimeric CS polymerase complexes[1,2]. Models are shown in cartoon representation with CHSY1 in green, CHPF in grey, CHSY3 in orange, and CHPF2 in light blue. The corresponding predicted aligned error (PAE) matrices are shown below each model. **(g)** Superposition of glycophorin A (PDB ID: 1AFO) with the CHSY3–CHPF complex model, with a zoom-in of the N-terminal region shown alongside. **(h)** Excerpt of the sequence alignment between glycophorin A, CHSY3, and CHPF, highlighting conservation of the GxxxG motif, which is frequently found in transmembrane coiled-coil interactions.

Supplementary Table 1: AlphaFold2 prediction scores.

The table summarizes the interface predicted template modeling (ipTM) values and predicted local distance difference test (pLDDT) values for AlphaFold2 predicted dimeric CS polymerase complexes.

**Complexes**	**pLDDT**	**ipTM**
CHSY1-CHSY1	77	0.292
CHPF-CHPF	82.9	0.69
CHSY3-CHSY3	77.8	0.3
CHPF2-CHPF2	76.6	0.18
CHSY1-CHPF	89.9	0.921
CHSY1-CHSY3	78.9	0.671
CHSY1-CHPF2	89	0.911
CHPF-CHSY3	89.9	0.919
CHPF-CHPF2	81.8	0.364
CHSY3-CHPF2	88.6	0.903

Supplementary Table 2: Mass photometry results for wild-type CS polymerase complexes.

Detailed summary of values obtained from the analysis of distinct peaks observed in mass photometry experiments, suggesting the presence of monomeric, dimeric, and tetrameric species.

	**Sample**	CHSY1-CHPF		CHSY1-CHPF2		CHSY3-CHPF		CHSY3-CHPF2	
	**Expected dimeric MW (kDa)**	166.3		169		165.1		167.8	
**1st peak**	**MW (kDa)**	90	80	61	-	62	67	82	95
	**σ (kDa)**	19.4	23	15.5	-	14.7	20	22	23
	**Counts**	286	251	64	-	72	127	376	400
	**Proportion (%)**	21	32	5	-	8	11	19	16
	**Mean MW (kDa) ± SD**	**85 ± 7.1**		**61**		**64.5 ± 3.5**		**88.5 ± 9.2**	
**2nd peak**	**MW (kDa)**	166	168	178	176	171	172	175	178
	**σ (kDa)**	15.1	18.7	18.6	15.2	19.3	14.5	31	31
	**Counts**	806	415	1279	2546	764	975	1581	2087
	**Proportion (%)**	59	53	94	89	88	85	81	84
	**Mean MW (kDa) ± SD**	**167 ± 1.4**		**177 ± 1.4**		**171.5 ± 0.7**		**176.5 ± 2.1**	
**3rd peak**	**MW (kDa)**	340	-	-	-	-	-	-	-
	**σ (kDa)**	20	-	-	-	-	-	-	-
	**Counts**	100	-	-	-	-	-	-	-
	**Proportion (%)**	7	-	-	-	-	-	-	-
	**Mean MW (kDa) ± SD**	**340**		-		-		-	

Supplementary Table 3: Mass photometry results for CHSY3–CHPF complexes under native and denaturing conditions.

Detailed summary of values obtained from the analysis of distinct peaks observed in mass photometry experiments following treatment of the CHSY3–CHPF complex with 5.4 M urea. No monomer peak was observed for CHSY3-CHPF in the absence of 5.4 M urea.

		CHSY3-CHPF (-Urea)		CHSY3-CHPF (+Urea)	
**monomer peak**	**MW (kDa)**	-	-	84	77
	**σ (kDa)**	-	-	13.1	11.6
	**Counts**	-	-	440	400
	**Proportion (%)**	-	-	57	59
	**Mean MW (kDa) ± SD**	-		**80.5 ± 4.9**	
**dimer peak**	**MW (kDa)**	162	167	153	149
	**σ (kDa)**	15	14.1	19.3	21
	**Counts**	92	1518	198	169
	**Proportion (%)**	2154	90	25	25
	**Mean MW (kDa) ± SD**	**164.5 ± 3.5**		**151 ± 2.8**	

Supplementary Table 4: EM data collection and refinement statistics.

**Data collection**	
Microscope	Titan Krios CM02 G4 (Thermo Fisher Scientific)
Voltage	300kV
Detector	Falcon 4i
Energy filter	Selectris X energy filter cold FEG
Image format	.tiff
Magnification	165,000 x
Defocus range (µm)	-2,1 to -0,5
Total exposure dose ($e^-/Å^2$)	50
Dose rate (e^-/px/s)	6.5
Raw pixel size (Å)	0.73
Spherical aberration (mm)	2.7
Nr frames and fractions	159 frames and 68 fractions
**Data processing**	
Software used for image processing	cryoSPARC v3.3.1
Number of movies collected	12,169
Number of good micrographs	7,598
Final number of particles	166,015
Box size (pix)	384
Symmetry imposed	No
FSC threshold	0.143
Map resolution (Å)	3.0
N-ter map resolution (Å)	2.9
C-ter map resolution (Å)	3.0
Local filtering with B factor	-40
Map visualizing software	ChimeraX
**Model building and refinement**	
PDB accession ID	9Q8Z
Initial model used	Alphafold 2 model
Model composition<br>Non-hydrogen atoms<br>Protein residues<br>Ligands	 9612<br>1179<br>2 MN; 1 UDP; 3 NAG
Root mean square deviations<br>Bond lengths (Å)<br>Bond angles (°)	 0.003<br>0.545
Ramachandran plot<br>Favored (%)<br>Allowed (%)<br>Disallowed (%)	 95.82<br>4.18<br>0
Validation<br>Molprobity score<br>Clashscore<br>Poor rotamers (%)	 1.8<br>9.79<br>0

Supplementary Table 5: Top 10 structures most similar to the CHSY3 middle domain in the Protein Data Bank (PDB90 - 90% sequence identity cut-off) based on DALI[7] search.

PDB-ID	Z-score	RMSD	%ID*	Match within the structure
**1YVB**	7.4	2.6	15	Cystatin from *Gallus gallus*
**5ML9**	7.3	2.9	11	Affimer F4 (synthetic construct)
**4IT7**	7.3	2.2	10	Cysteine protease inhibitor from *Ascaris lumbricoides*
**8V57**	7.3	3.5	18	Cystatin C from *Mus musculus*
**1RN7**	7.1	2.8	13	Cystatin D from *Homo sapiens*
**2BO9**	7.1	2.4	14	Latexin from *Homo sapiens*
**4N6M**	7	2.6	11	Cystatin M from *Homo sapiens*
**6UIO**	6.9	2.8	13	Cystatin-8 from *Mus musculus*
**3IMA**	6.8	2.2	15	Tarocystatin from *Colocasia esculenta*
**6STJ**	6.6	2.5	11	Cystatin (saltans group)

* RMSD: Root Mean Square Deviation, %ID: sequence identity (%)

Supplementary Table 6: Top 10 structures most similar to the CHPF middle domain in the Protein Data Bank (PDB90 - 90% sequence identity cut-off) based on DALI[7] search.

PDB-ID	Z-score	RMSD	%ID*	Match within the structure
**5ML9**	7.9	2.5	12	Affimer F4 (synthetic construct)
**6SAZ**	7.5	2.2	9	Astacin from *Astacus astacus*
**4LZI**	7.2	4.1	10	Multicystatin from *Solanum tuberosum*
**1WNH**	7.1	2.2	15	Latexin from *Mus musculus*
**3IMA**	7.1	1.8	10	Tarocystatin from *Colocasia esculenta*
**2W9P**	7	2.2	13	Multicystatin from *Solanum tuberosum*
**6Z0O**	6.8	1.9	12	Affimer-NP (synthetic construct)
**5OHM**	6.8	1.8	12	Polyubiquitin-C from *Homo sapiens*
**6STJ**	6.8	2.1	12	Cystatin (saltans group)
**6YR8**	6.7	1.9	10	Affimer K6 from Homo sapiens

* RMSD: Root Mean Square Deviation, %ID: sequence identity (%)

Supplementary Table 7: Top 10 structures most similar to the CHSY3-CHPF complex middle domain in the Protein Data Bank using Foldseek multimer[8]. Redundant results were omitted.

PDB-ID	Score	Prob.*	%ID*	Match within the structure
**2CH9**	43	0.28	10.1	Dimeric cystatin F from *Homo sapiens*
**6HPV**	47	0.38	10.1	Fetuin-B from *Mus musculus*
**1YVB**	64	0.85	16.9	Cystatin from *Gallus gallus*
**6SAZ**	28	0.08	13.2	Astracin from *Astacus astacus*
**8H2I**	23	0.04	12.9	P6 protein in PBCV-1 capsid from *Paramecium bursaria* Chlorella virus 1
**4N6N**	54	0.6	11.5	Cystatin E from *Homo sapiens*
**1NB3**	25	0.06	14.1	Stefin A from *Homo sapiens*
**6UIO**	36	0.15	15	Cystatin-8 from *Mus musculus*
**4IT7**	57	0.69	9	Cysteine protease inhibitor *Ascaris lumbricoides*
**3NX0**	37	0.16	15.6	Cystatin C from *Homo sapiens*

*** prob.: probability, %ID: sequence identity (%)**

Supplementary Table 8: Mass photometry results for mutant CHSY3-containing CS polymerase complexes.

Detailed summary of values obtained from the analysis of distinct peaks observed in mass photometry experiments, suggesting the presence of monomeric, dimeric, tetrameric, and hexameric species.

	Sample	D261N/ D263N		H394A		D718N/D720N		H831A	
1st peak	MW (kDa)	89	85	64	68	76	77	78	77
	σ (kDa)	21	27	20	15.4	25	19	24	23
	Counts	309	353	93	132	228	163	90	55
	Proportion (%)	19	24	10	8	20	20	5	4
	Mean MW (kDa) ± SD	**87 ± 2.8**		**66 ± 2.8**		**76.5 ± 0.7**		**77.5 ± 0.7**	
2nd peak	MW (kDa)	172	175	170	174	184	186	178	179
	σ (kDa)	29	24	16.4	26	30	19.6	15.3	18.6
	Counts	831	770	521	1062	594	423	993	778
	Proportion (%)	51	52	58	64	53	51	61	60
	Mean MW (kDa) ± SD	**173.5 ± 2.1**		**172 ± 2.8**		**185 ± 1.4**		**178.5 ± 0.7**	
3rd peak	MW (kDa)	357	373	357	362	387	391	365	367
	σ (kDa)	27	31	16.4	21	23	26	76	18.8
	Counts	277	197	138	281	160	89	402	243
	Proportion (%)	17	13	15	17	14	11	24	19
	Mean MW (kDa) ± SD	**365 ± 11.3**		**359.5 ± 3.5**		**389 ± 2.8**		**366 ± 1.4**	
4th peak	MW (kDa)	562	564	-	529	-	-	560	550
	σ (kDa)	109	42	-	48	-	-	32	19.9
	Counts	152	76	-	74	-	-	86	59
	Proportion (%)	9	5	-	4	-	-	5	5
	Mean MW (kDa) ± SD	**563 ± 1.4**		**529**		**-**		**555 ± 7.1**	

Supplementary Table 9: Summary of HEK293 cell clones generated with *CHPF/CHPF2* double gene knock-outs.

Bold letters in black denote insertions and bold letters in blue denote PAM sequences.

Gene	Clone	Sequence	Indels	Result
CHPF	WT	**CCG**GCTGGAGCGTGTGGTGTTCCTGACGGGCGCACGGGGCCGCCGGGCCCCACCTGGCATGGCAGTGGTGACGCTA		
	B2	**CCG**GCTG-A----GTGGTGTTCCTGACGGGCGCACGGGGCCGCCGGGCCCCACCTGGCATGGCAGTGGTGACGCTA	-5	fs
	E7	**CCG**GCTG-AGCGTGTGGTGTTCCTGACGGGCGCACGGGGCCGCCGGGCCCCACCTGGCATGGCAGTGGTGACGCTA	-1	fs
		**CCG**GC**GTTGTTTCTGTTGAGACAAACTCTCGCTCTATTCCTCAGCTGGTCATC**TGGAGCGTGTGGTGTTCCTGACG	+48	ns
	C1	**CCG**GCT**G**GGAGCGTGTGGTGTTCCTGACGGGCGCACGGGGCCGCCGGGCCCCACCTGGCATGGCAGTGGTGACGCT	+1	fs
	C2	----CTGGAGCGTGTGGTGTTCCTGACGGGCGCACGGGGGCCGCCGGGCCCCACCTGGCATGGCAGTGGTGACGCT	-36	Δ12 aa
		**CCG**GCT-GAGCGTGTGGTGTTCCTGACGGGCGCACGGGGCCGCCGGGCCCCACCTGGCATGGCAGTGGTGACGCTA	-1	fs
CHPF2	WT	**CCC**TCGGTTACTCTACTTCACTGGGCAGCGGGGGGCCCGGGCTCCAGCAGGGATGCAGGTGGTGTCTCATGGGGAT		
	B2	**CCC**TCG-TTACTCTACTTCACTGGGCAGCGGGGGGCCCGGGCTCCAGCAGGGATGCAGGTGGTGTCTCATGGGGAT	-1	fs
	E7	----------------------GGGCAGCGGGGGGCCCGGGCTCCAGCAGGGATGCAGGTGGTGTCTCATGGGGAT	-22	fs
		**CCC**TCGG**G**TTACTCTACTTCACTGGGCAGCGGGGGGCCCGGGCTCCAGCAGGGATGCAGGTGGTGTCTCATGGGGA	+1	fs
	C1	---CAGGTGGTGTCTCATGGGGAT	-60	unresolved
		**CCC**TCG-TTACTCTACTTCACTGGGCAGCGGGGGGCCCGGGCTCCAGCAGGGATGCAGGTGGTGTCTCATGGGGAT	-1	fs
	C2	**CCC**TCGGT-ACTCTACTTCACTGGGCAGCGGGGGGCCCGGGCTCCAGCAGGGATGCAGGTGGTGTCTCATGGGGAT	-1	fs

fs: frame shift, ns: nonsense mutation

Supplementary Table 10: Primer for cloning of expression constructs.

Mutation	Primer 5'- 3' sequence
**CHSY1_68_AgeI_for**	tat ACCGGT GCA CAA CTG TGG CCC CCA
**CHSY3_157_Age1_for**	tat ACCGGT TCA GGA GAT GGC GGA GCC
**CHPF_81_Age1_for**	tat ACCGGT GGC GAG AAT TGG GAG CCC
**CHPF2_57_AgeI_for**	tat ACCGGT AGC CGT GCC AGG CTG GAT
**CHSY1_802_KpnI_rev**	tat GGTACC AGC GGT GCG TAC ACT TCC
**CHSY3_882_Kpn1_rev**	tat GGTACC AGA TAA GGT CCG GTT ATA CCG
**CHPF_775_Kpn1_rev**	tat GGTACCGGT ACT GTT GCC CTG CTC C
**CHPF2_772_KpnI_rev**	tat GGTACC CGT ACT GTT AGC TTG TTC TTG

Supplementary Table 11: Primer for site-directed mutagenesis PCR.

Mutation	Forward primer (5’- 3’ sequence)	Reverse primer (5’- 3’ sequence)
**CHSY1 D171N/D173N**	GT GCC AAC GAC AAT GTC TAT ATT AAG GGC GAT AGA CTT GAA	GAC ATT GTC GTT GGC AC GCA TGA ACC ACT CGT ATT TAT CC
**CHSY1 H304A**	T ACT CTT GCT CCA AAT A AGA ATC CCC CAT ATC AAT ATC GA	T ATT TGG AGC AAG AGT A ATC GCC TGA TGT ATC TTG GAA
**CHSY1 D631N/D633N**	TTC TTT TGT AAT GTA AAT TTG GTC TTC ACA ACT GAG TTT CTG C	CAA ATT TAC ATT ACA AAA GAA CAA GAG GCT CTC GTT ATT GAA
**CHSY1 H744A**	C GTG GTT GCT GTT CAT CAC CCA GTG TTC TGC GAC C	ATG AAC AGC AAC CAC G CCC ACC TCT TGA CTT CTG AAT GTT
**CHSY3 D261N/D263N**	GG GCA AAT GAT AAC GTGTAT ATA AAG GGC GAC AAG CTT GA	CAC GTT ATC ATT TGC CC TCA TAA ACC ATT CGT ACT TAT CGA G
**CHSY3 H394A**	ACG CTG GCC CCT AAT AAG CGC CCA GCC TAT CAG TA	ATT AGG GGC CAG CGT AAT TGC GGC GTG AAT CTT AG
**CHSY3 D718N/D720N**	TTC TGC AAC GTA AAT CTG ATC TTT CGG GAA GAC TTC TTG C	CAG ATT TAC GTT GCA GAA CAG AAG CAG GGT ATC GTT ATC G
**CHSY3 H831A**	GT GTA GTT GCC ATC TTT C ACC CCG TGC ACT GCG AC	GAA AGA TGG CAA CTA CAC CCA CCT CTT GGG ACC GA

References

1. Jumper, J. *et al.* Highly accurate protein structure prediction with AlphaFold. *Nature* **596**, 583–589 (2021).
2. Mirdita, M. *et al.* ColabFold: making protein folding accessible to all. *Nat Methods* **19**, 679–682 (2022).
3. Varki, A. *et al.* Symbol Nomenclature for Graphical Representations of Glycans. *Glycobiology* **25**, 1323–1324 (2015).
4. Meng, E. C. *et al.* UCSF ChimeraX: Tools for structure building and analysis. *Protein Science* **32**, e4792 (2023).
5. Madeira, F. *et al.* The EMBL-EBI Job Dispatcher sequence analysis tools framework in 2024. *Nucleic Acids Res* **52**, W521–W525 (2024).
6. Robert, X. & Gouet, P. Deciphering key features in protein structures with the new ENDscript server. *Nucleic Acids Research* **42**, W320–W324 (2014).
7. Holm, L. Dali server: structural unification of protein families. *Nucleic Acids Res* **50**, W210–W215 (2022).
8. Kim, W. *et al.* Rapid and sensitive protein complex alignment with Foldseek-Multimer. *Nat Methods* **22**, 469–472 (2025).

Uncropped gels and blots

Supplementary Figure 1a

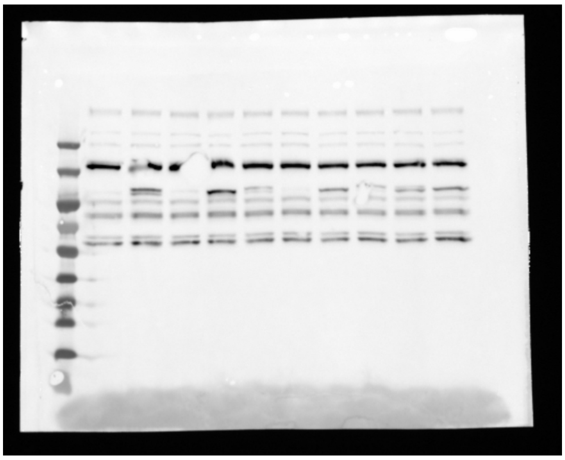

Supplementary Figure 1b

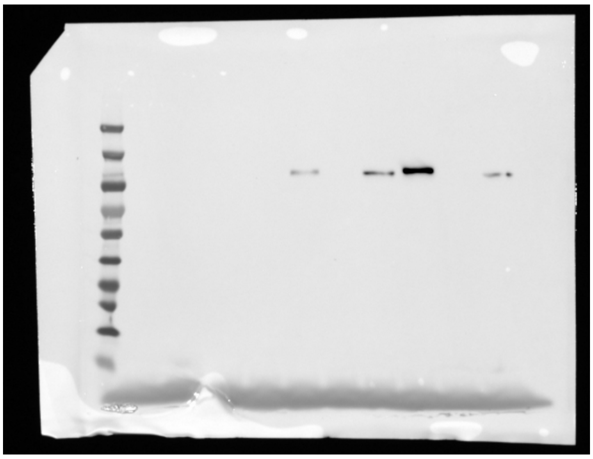

Supplementary Figure 1c

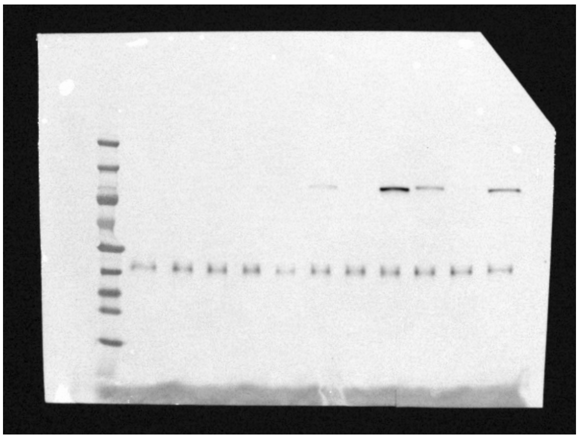

Supplementary Figure 1d

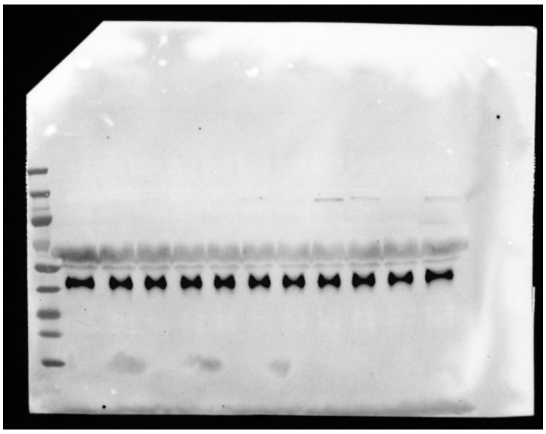

Supplementary Figure 4a

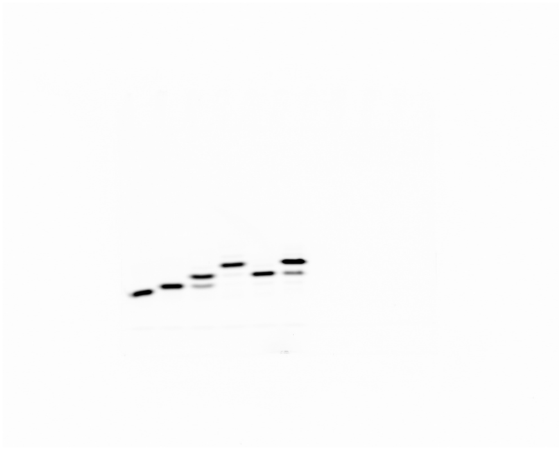

Supplementary Figure 4c

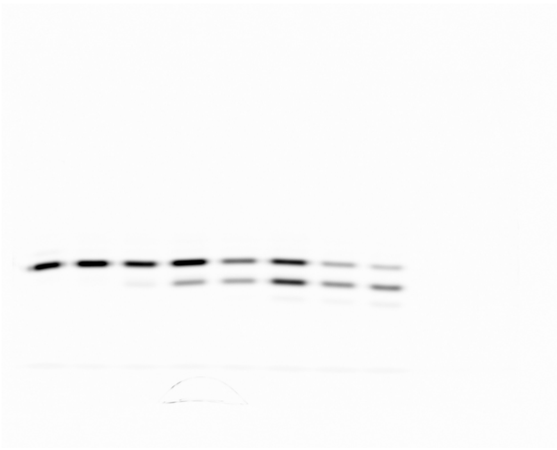

Supplementary Figure 12a

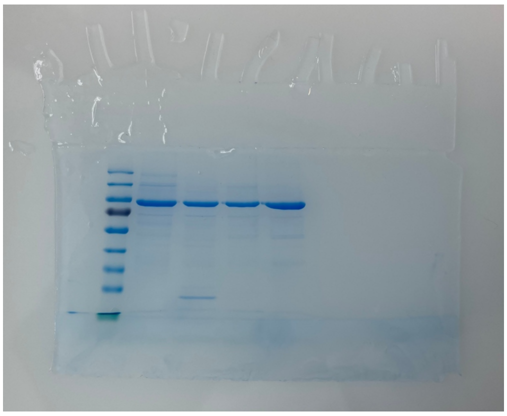

Supplementary Figure 14a

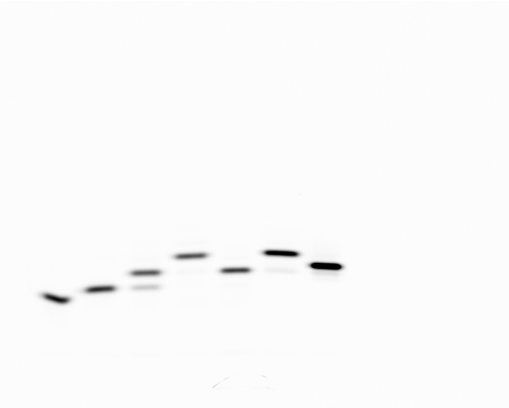

Supplementary Figure 14c

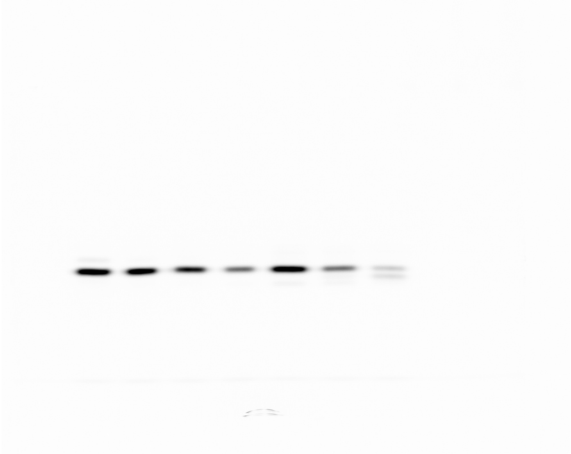

Supplementary Figure 15-1

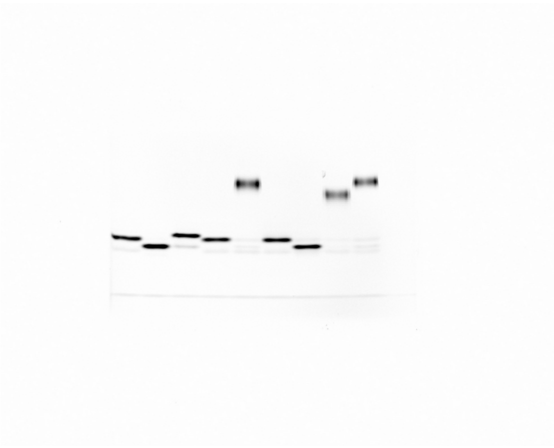

Supplementary Figure 15-2

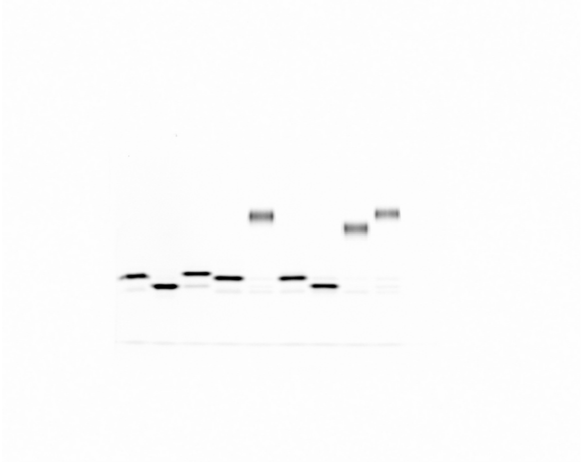

Supplementary Figure 15-3

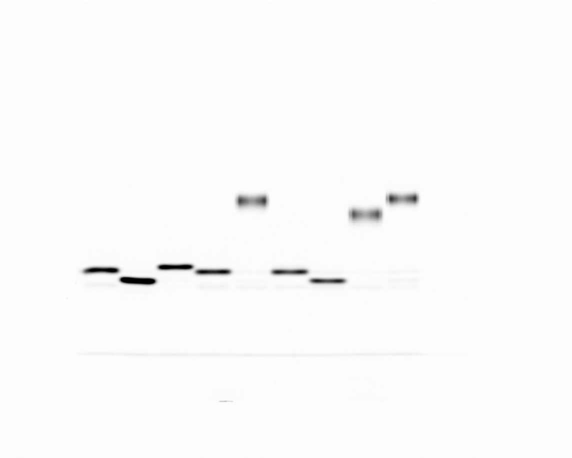

Supplement: Supplementary file 1 — Supplementary Information [file 41467_2025_66787_MOESM1_ESM.pdf]
